# Impact of Wedge Parameters on Ultrasonic Lamb Wave Liquid-Level Sensor

**DOI:** 10.3390/s22135046

**Published:** 2022-07-04

**Authors:** Weizhao Xue, Wanjia Gao, Wenyi Liu, Huixin Zhang, Ruiqing Guo

**Affiliations:** 1 Key Laboratory of Instrumentation Science & Dynamic Measurement, Ministry of Education, North University of China, Taiyuan 030051, China; s2006112@st.nuc.edu.cn (W.X.); b1806014@st.nuc.edu.cn (W.G.); liuwenyi@nuc.edu.cn (W.L.); 2 Guangzhou Institute of Technology, Xidian University, Guangzhou 510555, China; rqguo@stu.xidian.edu.cn

**Keywords:** ultrasonic Lamb waves, liquid-level measurement, sound field, wedge block

## Abstract

The ultrasonic Lamb wave detection principle can realize the noncontact measurement of liquid level in closed containers. When designing an ultrasonic Lamb wave sensor, it is vital to thoroughly study and select the optimal wedge size at the front of the sensor. In this paper, firstly, we select the best working mode of Lamb waves according to their propagation dispersion curve in aluminum alloy, and we obtain the best angle of wedge through experiments. Secondly, we study the impact of the size of the wedge block on the results, and we obtain the selection method of wedge block parameters. The evaluations show that, when the frequency–thickness product is 3 MHz·mm, the Lamb waves work in the A1 mode, and the experimental effect is the best. At this time, the incident angle of the ultrasonic wave is 27.39°. The wedge thickness should be designed to avoid the near-field area of the ultrasonic field, and we should choose the length as odd multiples of 1/4 wavelength. The rules obtained from the experiment can effectively select the best working mode for ultrasonic Lamb waves, while also providing a basis for the design of the wedge block size in a Lamb wave sensor.

## 1. Introduction

In the fields of food safety, industry, and aviation, it is very necessary to monitor liquids in closed containers under harsh environments [1]. Therefore, the research and development of nondestructive testing (NDT) sensors is important.

At present, liquid-level sensors mainly include capacitive, resistive, float, radar, laser, acoustic, and fiber-optic sensors [2,3,4,5]. Capacitive, resistive, float, and fiber-optic sensors are all contact sensors, which are not suitable for large, closed containers and cannot achieve NDT. Although radar-type and laser-type sensors are not directly in contact with the measured liquid, they need to be installed inside the container, which destroys the integrity of the container and is expensive. The acoustic-based detection method can realize true noncontact measurement outside the tank [6] and is, therefore, best suited for this application.

Commonly used acoustic wave-based liquid-level detection techniques include the interface reflection method [7], penetrative method [8], and attenuation method [9]. Since the attenuation of the ultrasonic guided wave along the propagation path is very small, it can even travel a very long distance along with the detected object [10]. At the same time, ultrasonic waves can result in particle vibration on the upper and lower surfaces of the plate, as well as in the middle part, and the sound field can cover the whole detected structure [11]. Therefore, ultrasonic guided wave detection technology can overcome many shortcomings of traditional methods [12]. It is more suitable for the detection of liquid level in large containers such as space launch vehicle fuel tanks.

Lamb waves are a type of guided waves. During the propagation process, they carry information such as the group velocity, phase velocity, and amplitude attenuation related to material properties, waveguide thickness, and propagation frequency. They have been widely used in various sensors and liquid-level detection [13]. Spratt et al. [14] proposed a new intrusive torsional waveguide sensor for temperature and liquid-level measurement. The variation of the flight time of the torsional wave in the waveguide can be related to the temperature and liquid level. Balasubramaniam et al. [15] described noninvasive ultrasonic guided wave systems for fluid-level sensing, and presented the finite element modeling, design, and construction of waveguide sensors for fluid-level sensing. Lingyu et al. [16] presented a case study of guided waves in a steel plate with one side immersed in water. They proposed that the water level affects the wave propagation time linearly and can be potentially used for estimation of water level in a container.

Although Lamb waves have the above advantages, their application is limited due to the complex dispersion relationship and multimode characteristics. The detection effect and detection accuracy of ultrasonic Lamb waves are determined by whether the corresponding dispersion curve can be quickly and accurately obtained [17]. Zamanov et al. [18] used the three-dimensional linearized theory of propagation of elastic waves in bodies with initial stresses and investigated the propagation of Lamb waves in this plate. Kim et al. [19] developed a hybrid one-dimensional finite element (1D FE) analytical method to analyze the Lamb wave propagation characteristics of composite panels. It allows using a small number of finite elements even for high-frequency analysis in a computationally efficient manner. Wilcox and Lowe et al. [20] conducted a study on excitation modes and sensors for long-range Lamb wave detection, pointed out that six factors should be considered in the selection of Lamb wave modes and frequencies, which are dispersion, attenuation, sensitivity, excitability, detectability, and selectivity.

Although many researchers have conducted a large number of studies on the propagation characteristics of Lamb waves and liquid-level detection applications using Lamb waves, it is difficult to find specific research results on internal wedge parameters in making Lamb wave sensors. The wedge is an essential component in the Lamb wave sensor, as it can act as a buffer block to avoid the near-field area [21]. Liu et al. [22] compared the materials of the buffer block in detail and drew the curve of the sound velocity with frequency and temperature in PMMA. They combined other physical properties of PMMA, and finally proposed that PMMA is the most suitable material in the measurement experiment of liquid acoustic properties. Nikolaevtsev et al. [23] investigated Lamb wave excitation by a wedge-shaped ultrasonic phase array transducer using the finite element (FEM) method. They investigated the influence of three different liquid layers and the dependence on the aperture of the transducer.

In conclusion, on the basis of the principle that ultrasonic Lamb waves can propagate in thin walls, this paper builds an external fixed-point noncontact liquid-level monitoring system. In this method, we use a wedge with a certain inclination angle, so that Lamb waves can be generated after ultrasonic waves are incident. The Lamb waves propagate in the container wall, and the residual waves are received by the receiving device. Firstly, we draw the dispersion curve of Lamb waves according to the properties of the experimental device material, select the optimal working mode of Lamb waves required for the experiment, and then verify its feasibility by experiments with wedges with different inclination angles. Secondly, we study the impact of wedge thickness on the experiment and select the best wedge size available for the experiment. On this basis, we study the impact of the wedge size on the results when there is a multiple relationship between the size of the wedge and the wavelength of ultrasonic wave. The general rules summarized in this paper provide a powerful basis for the selection of the best working mode of Lamb waves and the design of wedge size in NDT based on ultrasonic Lamb waves.

## 2. Theory and Methods

### 2.1. Lamb Wave Dispersion Characteristics

Lamb waves propagate in a structure with two parallel surfaces and are a stress wave formed by the coupling of shear waves (*s*-waves) and longitudinal waves (*p*-waves) [24]. Lamb waves are guided waves propagating in a plate, also known as plate waves. The dispersion curve of Lamb waves is the main basis for selecting appropriate modes and frequencies for plate structures of different materials [25]. The dispersion characteristics of Lamb waves are described by the Rayleigh–Lamb equation [26].

The symmetric mode (S Mode) is

(1) $$\frac{\tan(qd)}{\tan(pd)}=-\frac{4k^{2}pq}{{\left(q^{2}-k^{2}\right)}^{2}}.$$

The antisymmetric mode (A Mode) is

(2) $$\frac{\tan(qd)}{\tan(pd)}=-\frac{{\left(q^{2}-k^{2}\right)}^{2}}{4k^{2}pq}.$$

Here,

(3) $$p^{2}=\frac{\omega^{2}}{c_{L}^{2}}-k^{2};\;q^{2}=\frac{\omega^{2}}{c_{T}^{2}}-k^{2};\;k=\frac{\omega }{c_{P}}=\frac{2\pi f}{c_{P}},$$

where *c_L_* is the *p*-wave velocity (m/s), *c_T_* is the *s*-wave velocity (m/s), *k* is the wavenumber, *f* is the frequency of Lamb waves (Hz), *c_p_* is the phase velocity (m/s), and *d* is 1/2 of the thickness of the measured structure (m). *c_L_* and *c_T_* can be calculated using Equations (4) and (5).

(4) $$c_{L}=\sqrt{\frac{E(1-v)}{\rho (1+v)(1-2v)}},$$

(5) $$c_{T}=\sqrt{\frac{E}{2\rho (1+v)}},$$

where *E* is Young’s modulus of the tested structure (GPa), *ρ* is the density of the material of the structure (g/cm^3^), and *v* is the Poisson’s ratio of the structure. The group velocity *c_g_* can be obtained from the phase velocity *c_p_* [17], which can be calculated using Equation (6).

(6) $$c_{g}=\frac{d\omega }{dk}.$$

Therefore, the *p*-wave velocity and *s*-wave velocity of the Lamb waves can be calculated according to the Young’s modulus, density, and Poisson’s ratio of plate material. Then, the plate Lamb wave dispersion curve can be drawn accordingly.

Ultrasonic waves at different incidence angles will result in different wave modes. The incident angle *α*, the phase velocity *c_p_*, and the ultrasonic wave velocity *c*_*L*1_ in the wedge satisfy Snell’s theorem [27], which can be calculated using Equation (7).

(7) $$\frac{\sin\alpha }{c_{L1}}=\frac{\sin90^{{}^{\circ}}}{c_{p}}.$$

### 2.2. Lamb Wave Sound Field Characteristics

When the ultrasonic waves are incident vertically as *p*-waves, due to the wave interference, the sound pressure fluctuates up and down on the axis near the wave source, and there are several maximum and minimum values. Acoustically, this area is called the near-field area of the ultrasonic, denoted by *N*. From the last maximum, the sound pressure decreases monotonically with the increase in distance. This area is called the far-field area of the ultrasonic [28]. The distribution of maximum and minimum values in the near field is dense. With the increase in distance, the distance between the maximum and minimum becomes wider [29]. The ultrasonic near-field area *N* can be calculated using Equation (8).

(8) $$N=\frac{D_{S}{}^{2}-\lambda^{2}}{4\lambda }\approx \frac{D_{S}{}^{2}}{4\lambda }=\frac{F_{S}}{\pi \lambda },$$

where *D_S_* is the ultrasonic sensor diameter (m), *F_S_* is the sensor area (m^2^), and *λ* is the wavelength of ultrasonic wave propagation in the medium. It can be obtained that the near-field length of the ultrasonic field is inversely proportional to the wavelength and directly proportional to the sensor area. A higher ultrasonic frequency results in a shorter wavelength and a longer near-field length.

**Figure 1:**
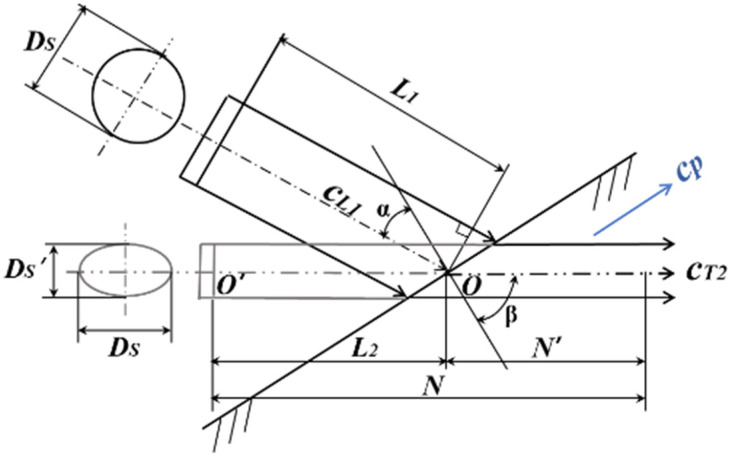
Lamb wave sound field.

When the *p*-waves are incident on the interface obliquely, the waveform is converted. The transmitted waves are divided into *s*-waves and *p*-waves. At this time, the sound field is composed of the *p*-wave sound field in the first medium and the *s*-wave sound field in the second medium. The two parts are broken. We can project the *p*-wave source in the first medium into a hypothetical *s*-wave source that coincides with the axis of the second medium. The sound field can be regarded as a continuous *s*-wave sound field [30]. Lamb waves are *p*-waves transmitted into the thin plate, which propagate along the direction of the parallel plate surface. The propagation velocity is its phase velocity *c_p_*. A schematic diagram of the Lamb wave sound field is shown in Figure 1.

In Figure 1, *α* is the *p*-wave incidence angle, and *β* is the *s*-wave refraction angle. They satisfy Equation (9).

(9) $$\frac{c_{L1}}{\sin\alpha }=\frac{c_{T2}}{\sin\beta },$$

where *c*_*L*1_ is the incident wave velocity of the *p*-waves, and *c*_*S*2_ is the *s*-wave velocity in the second medium. The near-field length *N* is calculated from the hypothetical wave source *O′*. It satisfies Equation (10).

(10) $$N=\frac{F_{s}}{\pi \lambda_{T2}}\frac{\cos\beta }{\cos\alpha }.$$

According to the above theory, the length of the near field of a Lamb wave is the same as the *p*-wave sound field, which is inversely proportional to the wavelength and directly proportional to the area of the wave source. The length *N′* of the near-field region in the second medium can be deduced from Equation (11).

(11) $$N^{\prime }=N-L_{2}=\frac{F_{3}}{\pi \lambda_{T2}}\frac{\cos\beta }{\cos\alpha }-L_{1}\frac{\mathrm{tg}\alpha }{\mathrm{tg}\beta },$$

where *λ_T_*_2_ is the *s*-wave wavelength in the second medium, *L*_1_ is the distance from the incident point to the wave source, and *L*_2_ is the distance from the incident point to the hypothetical wave source.

Therefore, we add a wedge block between the ultrasonic probe and the container wall. First, the angle of the wedge block can make the ultrasonic wave incident and get the desired Lamb wave working mode. Second, the wedge block can be a delay block, which can make the ultrasonic work in its far-field area. Therefore, the angle and thickness of the wedge are important factors in the experiment.

For the selection of wedge material, the acoustic impedance of the polymer is close to that of water, which makes the sensor more sensitive [31]. Therefore, in this paper, we chose polymethyl methacrylate (PMMA) [32]. PMMA wedges with different parameters used in this research were processed with a digitally controlled laser cutting machine (BL6050, G. Weike, Jinan, China), with a process accuracy ≤0.01 mm.

### 2.3. Establishment of Experimental Platform

On the basis of the above theory, this paper builds an external fixed-point liquid level NDT system based on ultrasonic Lamb waves. The material of the tested container is aluminum alloy, which is the same as the commonly used liquid storage tank material for space rockets. The internal measured medium is air and water. We use a continuous sine wave with amplitude of ±15 V to excite the transducer to produce ultrasonic waves. The wedge of a certain angle is used to generate the Lamb wave working mode required for the experiment. We use an ultrasonic couplant to closely fit the wedge to the wall of the container and the ultrasonic transducer, so as to discharge air, allowing the ultrasonic wave to be maximally transmitted into the container.

Two ultrasonic transducers are installed symmetrically on the outside of the container, one is the transmitter and the other is the receiver. The *p*-waves are emitted to the container at a certain angle, and, after penetrating the container, a Lamb wave propagating along the container wall is generated. Some of the waves produce secondary transmission at the interface between the inner wall of the container and the inner medium. According to the ultrasonic impedance method, the ability to reflect and transmit ultrasonic waves from different media is related to the difference in the acoustic impedance of materials. The sound intensity reflectance *R* and sound intensity transmittance *T* satisfy the following equations [33]:

(12) $$R=\frac{{\left(Z_{2}-Z_{i}\right)}^{2}}{{\left(Z_{2}+Z_{i}\right)}^{2}},$$

(13) $$T=\frac{4Z_{2}Z_{i}}{{\left(Z_{2}+Z_{i}\right)}^{2}},$$

where *Z*_2_ is the acoustic impedance of the tested container (Mrayl), and *Z_i_* is the acoustic impedance of the inner medium (Mrayl). In this paper, the material of the tested container is aluminum alloy, *Z*_2_ = 32 Mrayl, and the inner medium is water and air; the acoustic impedance of water is *Z_W_* = 1.48 Mrayl, and the acoustic impedance of air is *Z_A_* = 4 × 10^−4^ Mrayl. By substituting the above parameters into Equations (12) and (13), it can be obtained that the transmittance of water and air is *R_W_* = 83.10% and *R_A_* = 99.99%, respectively [32]. Therefore, water has less reflectivity and greater transmissivity than air.

**Figure 2:**
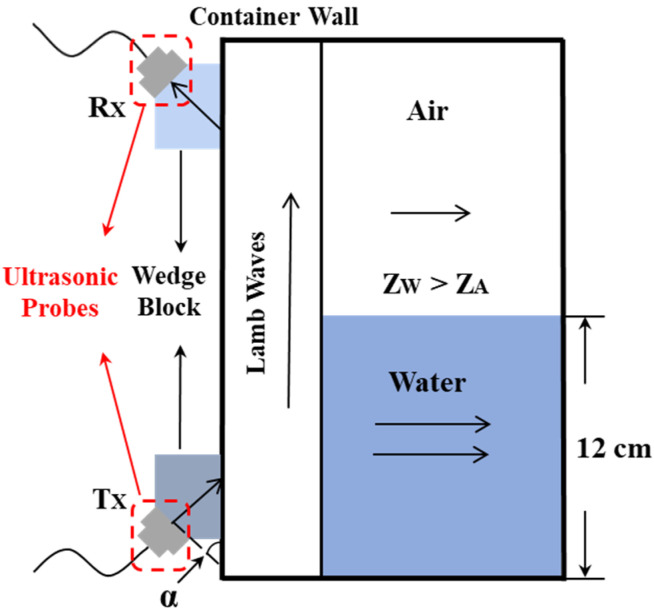
Schematic diagram of liquid-level measurement with ultrasonic Lamb waves. *R_x_* = receiver transducer; *T_x_* = transmitter transducer; *α* = ultrasonic incidence angle; *Z_W_* = water acoustic impedance; *Z_A_* = air acoustic impedance.

When there is liquid between the two transducers, the liquid absorbs part of the ultrasonic waves and forms a leaky Lamb wave. The remaining ultrasonic energy delivered to the receiver is reduced. There is a certain relationship between residual energy and liquid level. According to this relationship, we can get the liquid level corresponding to the received energy [17]. S schematic diagram is shown in Figure 2. This liquid-level measurement method can measure different level heights between two ultrasonic probes and does not need to consider the acoustic interference and attenuation inside the liquid, which is suitable for large containers. Therefore, the experimental platform built in this paper can replace the measurement environment of the rocket tank and meet the requirements of its liquid level measurement. It is also suitable for liquid-level measurement in other industrial fields. This paper focuses on wedge parameters, and we will specifically measure the liquid level in the future.

In this paper, the ultrasonic probe is made of piezoelectric ceramic (PZT), with a diameter of 10 mm, natural frequencies of 1 MHz and 500 kHz, and an emitted wave amplitude of ±15 V. The wall thickness of the measured container is 3 mm. The experimental measurement temperature is 20 °C. The experimental platform was built according to the above principles. The relevant initial values of the experimental device and the measured liquid are shown in Table 1.

The working process of the system is as follows: the ultrasonic wave is excited by the transmitter; then, the ultrasonic wave enters into the container wall and propagates in the wall in the form of Lamb waves. After wave transmission and attenuation in the wall, the remaining ultrasonic energy is received by the transducer at the receiving end. The receiver is connected to an oscilloscope (TDS 1001B, Tektronix, Shanghai, China), and the results are collected. Finally, we draw and analyze the result data on a computer.

## 3. Results and Discussion

On the basis of the above principles and devices, this paper conducted experiments in groups to explore the selection of the best working mode of the Lamb waves and the impact of wedge thickness on the results.

### 3.1. Selection of Lamb Wave Working Modes

**Figure 3:**
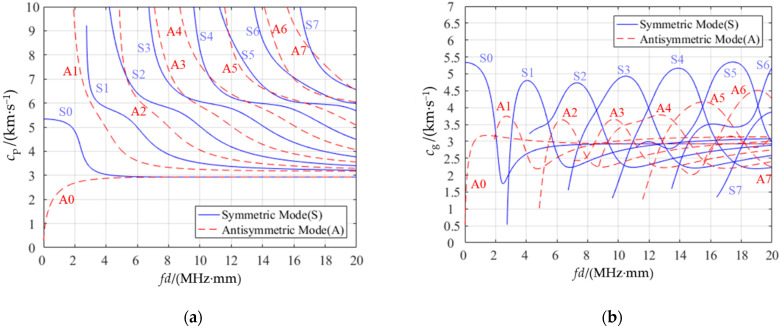
Dispersion curves of aluminum alloy: (**a**) phase velocity dispersion curve; (**b**) group velocity dispersion curve.

First, according to the Young’s modulus *E*, density *ρ*, and Poisson’s ratio *v* of the aluminum alloy in Table 1, we could obtain the *p*-wave velocity *c_L_* = 5907.6 m/s and the *s*-wave velocity *c_T_* = 3157.8 m/s of the Lamb waves propagating in thin-wall aluminum alloy. Then, we drew the dispersion characteristic curve of Lamb waves with MATLAB according to Equations (1)–(6), as shown in Figure 3a,b.

There are several principles for selecting the best working mode of Lamb waves. Firstly, to overcome the problem of multimode, it is best to choose a point farther away from other modes. Secondly, it is optimal to choose a situation where the group velocity curve is flat, whereby the group velocity changes less, and the echo distance is relatively stable [34].

As can be seen from Figure 3, dispersion curves of different materials were drawn according to ultrasonic frequency and material wall thickness. As shown in Table 1, the ultrasonic frequency *ƒ* = 1 MHz and the container wall thickness *d* = 3 mm; thus, the experimental intermediate frequency–thickness product *ƒd* = 3 MHz·mm. It can be seen from Figure 3a that 3 MHz·mm was available for four modes, namely, S0, S1, A0, and A1. However, the phase velocities of the S0 mode and A0 mode were too close to be suitable for selection. From Figure 3b, we chose a mode with a relatively flat group velocity curve, i.e., A1, for the Lamb waves selected in this experiment.

For other materials or measured objects with different wall thicknesses, the dispersion curve was still drawn according to the parameters of the materials. Then, the working mode was selected according to the frequency–thickness product and the principle mentioned above.

From Figure 3a, when *ƒd* = 3 MHz·mm, the corresponding Lamb wave phase velocity *c_p_* in A1 mode was 6 mm/us. According to Table 1, *c_L_*_1_ = 2775 m/s; substituting it into Equation (7), we could obtain the incident angle of the ultrasonic wave according to the Snell theorem, i.e., *α* = 27.39°.

**Figure 4:**
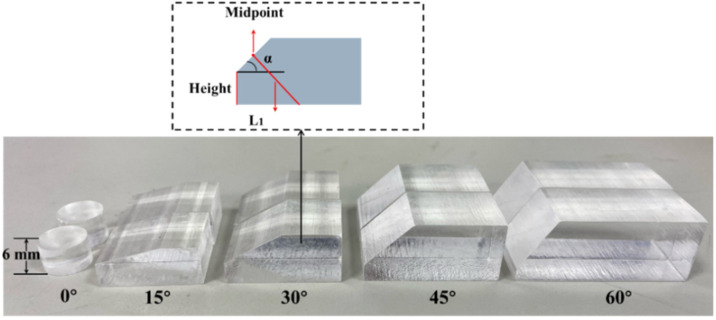
Physical images of PMMA wedges.

When *p*-waves are obliquely incident on the interface between two media, a first critical angle and a second critical angle are generated. The first critical angle is the incident angle when the refracted *p*-wave is 90°, and the second critical angle is the incident angle when the refracted *s*-wave is 90°. According to the *p*-wave and *s*-wave velocities of aluminum alloy (*c_L_*_2_ = 5907.6 m/s and *c_T_*_2_ = 3157.8 m/s) and the sound velocity of ultrasonic waves in PMMA (*c_L_*_1_ = 2775 m/s), we could calculate the first critical angle *α*_1_ = 28° and the second critical angle *α*_2_ = 61.45°. Therefore, the incident angle could not be greater than 61.45°; otherwise, there would be no ultrasonic waves in the second medium. To verify the correctness of the selection method proposed in this paper, we made five different wedges from PMMA of the same thickness, with dip angles of 0° (normal incidence), 15°, 30°, 45°, and 60°. Due to machining precision, the error of the wedge angle was within ±3°. There were two wedges of each type, used at the transmitter and receiver, as shown in Figure 4.

**Figure 5:**
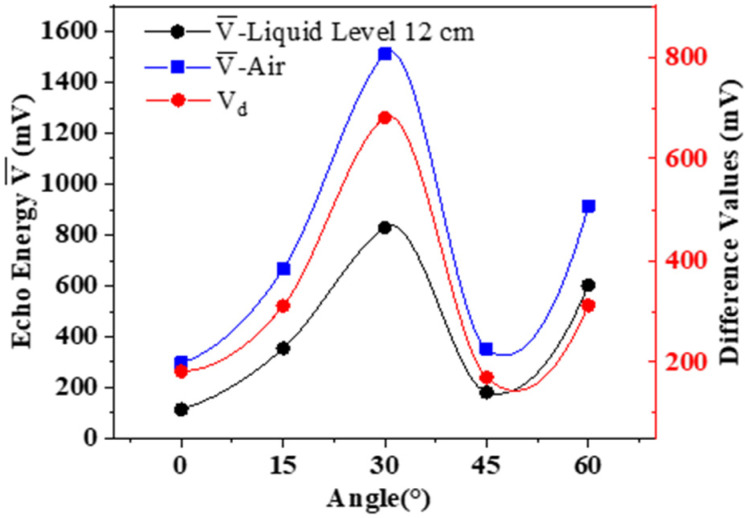
The impact of different ultrasonic incident angles on the experiment.

Under the same external conditions, we placed the wedges with different angles to detect the received ultrasonic energy when the liquid level was lower than the two sensors (air) and between the two sensors (the same height at each time, both at 12 cm ± 1 mm). Experiments were performed three times in each group. We calculated the average values $\bar{V}$ and compared the difference values *V_d_* of the ultrasonic energy at different liquid levels. The experimental data are shown in Table 2, and the evaluations were drawn as shown in Figure 5.

According to the above experimental data, under the same other conditions, when the wedge inclination was 30° (where the Lamb waves worked in the A1 mode), the ultrasonic residual energy received by the receiver was the largest regardless of the liquid level. Moreover, the energy difference between the presence and absence of water was also the most obvious, and the experimental effect was the best. When *α* = 0°, the received energy was the lowest, because, at this time, the transmitted wave was perpendicular to the incident side, most of the acoustic wave energy was reflected vertically, and very little energy propagated along the container wall to the receiver. The average difference of the experiment was no more than 50 mV. Therefore, it was verified that the method of optimal working mode selection of Lamb waves obtained in this paper was effective.

As another detection approach based on ultrasonic Lamb waves, we can select the best working mode of Lamb waves according to the method proposed in this paper, so as to determine the ultrasonic incident angle and to achieve the best detection effect.

### 3.2. Selection of Wedge Thickness

According to Table 1, when *T* = 20 °C, the ultrasonic sound velocity in the PMMA wedge *c_L_*_1_ = 2775 m/s, *f* = 1 MHz, and sensor diameter *D_S_* = 10 mm; substituting them into Equation (8), we could obtain that the length of the near-field area of *p*-waves *N* = *L*_1_ at this time was 9 mm, and the wavelength was 2.775 mm. By substituting *α* = 30°, *c_L_*_1_ = 2775 m/s, and *c_T_*_2_ = 3157.8 m/s into Equation (9), we could obtain the *s*-wave refraction angle *β* = 34.7°. Substituting them in Equation (10), we could get *N* = 7.50 mm. From Equation (11), we could get *N′* = 0.03 mm, which we could ignore. At this time, there was almost no *s*-wave near-field area in the second medium. The refraction angle of the Lamb waves was 90°. From Equations (10) and (11), we could get *N* = 0, and *N′* = −*L*_2_. That is, when we analyzed the near field of the Lamb sound field, only the near field of the incident *p*-waves could be considered.

For the design of the wedge’s long side, it was only necessary to ensure that the energy generated by PZT could be directly emitted to the bottom surface of the wedge (the contact surface between the wedge and the measured object), but not to the vertical surface of the wedge to cause the reflection of waves in the wedge.

After calculation, when the wedge thickness (height) was 0, the long side was at least 11.54 mm. The long side increased with the increase in thickness. In the experiment, the maximum height was 12.21 mm, and the long side was at least 18 mm. Therefore, the selection of the long sides had little effect on the energy collection. We made some wedges from PMMA with the same width of 20 mm, the same dip angle of 30°, and different thicknesses. The dimensions of the wedges are shown in Table 3, and some descriptions are shown in Figure 4.

**Figure 6:**
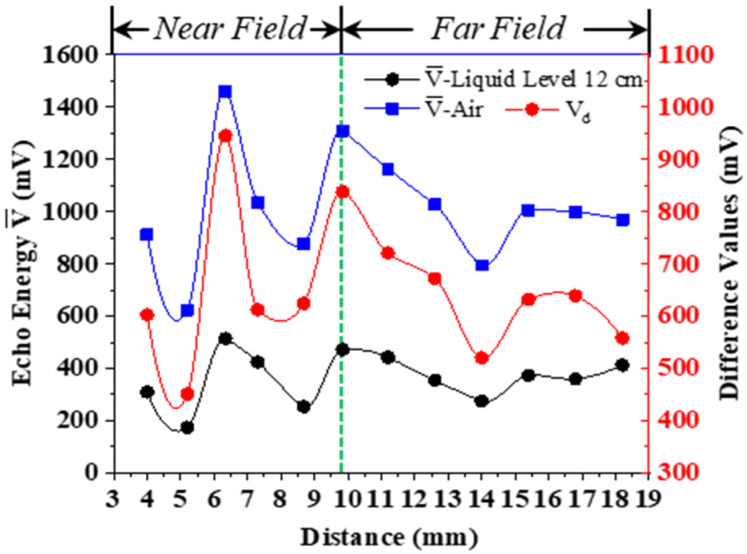
The impact of ultrasonic field on experimental results.

Under the same external conditions, we replaced the wedges with different thickness to detect the received ultrasonic energy when the liquid level was lower than the two sensors (air) and between the two sensors (the same height at each time, both at 12 cm ± 1 mm). Experiments were performed three times in each group. We calculated the average values $\bar{V}$, and we compared the difference values *V_d_* of ultrasonic energy at different liquid levels. The evaluations were drawn as shown in Figure 6.

According to the above evaluations, when the emitted ultrasonic wave reached the container wall and was still in the near-field region of the ultrasonic field, the received wave had some maximum and minimum values. The distance between the two minimum values was relatively close. When the transmitting distance just avoided the near-field area, the received echo amplitude was larger. As the distance of the transmitted wave increased, the amplitude tended to decrease. The average difference in the experiment was no more than 20 mV. However, the amplitude change tended to be flat, and it was not easy to see the rule and draw conclusions. Therefore, the experiment described in Section 3.3 was performed.

### 3.3. Impact of Wavelength on Experiments

In the experiment conducted in Section 3.2, since the distance (*L*_1_) of the ultrasonic wave from emission to the container wall was exactly an integer multiple of the half-wavelength of the sound wave, the measured point could have been placed at the node between the crest and trough of the sound wave. The result of this measurement tended to be a straight line, and no certain rule could be obtained.

To study the impact of the wavelength of the ultrasonic wave on the evaluations, we reduced the frequency of the emitted wave from 1 MHz to 500 kHz. When the sound velocity was constant, the frequency decreased by half, and its wavelength doubled. At this time, the wavelength *λ* was 5.55 mm. Therefore, *L*_1_ was an integer multiple of a quarter-wavelength of ultrasound. The relationship between wedge size and wavelength is shown in Table 4.

**Figure 7:**
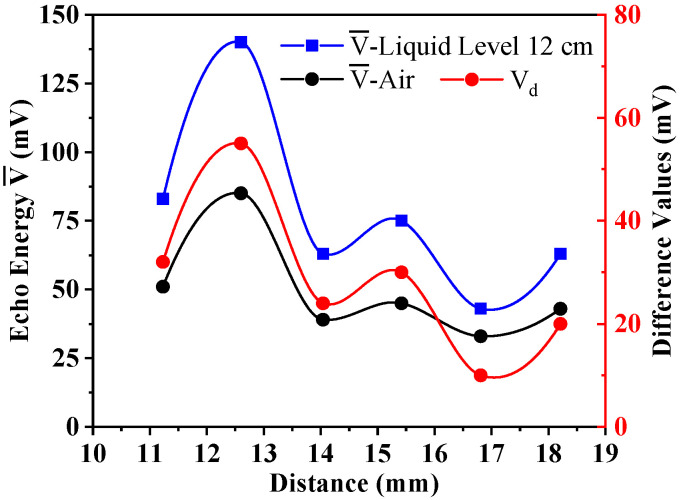
The impact of ultrasonic wavelength on experimental results.

Under the same external conditions, we replaced the wedges with different thickness to detect the received ultrasonic energy when the liquid level was lower than the two sensors (air) and between the two sensors (12 cm ± 1 mm). Experiments were performed three times in each group. We calculated the average values $\bar{V}$ and compared the difference values *V_d_* of ultrasonic energy at different liquid levels. The evaluations were drawn as shown in Figure 7.

As can be seen from the above results, when the propagation distance of the transmitted wave through the buffer block to the container wall was an even multiple of a quarter-wavelength, the received ultrasonic energy was lower, and it existed at the trough of the sound wave. When the distance was an odd multiple of a quarter-wavelength, the received ultrasonic energy was higher, and it existed at the peak of the sound wave. Furthermore, its energy gradually weakened with the increase in distance, conforming to the experimental law obtained in the far-field area in Figure 7. The average difference in the experiment was no more than 4.44 mV.

Therefore, combining the evaluations of Section 3.2 and Section 3.3, it can be concluded that, to ensure the most ideal experimental effect, when designing the wedge at the front end of the ultrasonic sensor, the vertical distance between the probe and the container wall should be just longer than the distance in the near field. Furthermore, the vertical distance should be an odd multiple of the quarter-wavelength of the ultrasonic wave, while even multiples should be avoided to transmit as many ultrasonic waves as possible into the measured container. In this experiment, when the emitted wave frequency was 1 MHz, the near field was just avoided when the distance was 9.84 mm. When the emitted wave frequency was 500 kHz, the experimental effect was best when the distance was 12.6 mm. At this time, the distance was just an odd multiple of a quarter-wavelength. To reduce the size of the wedge and to find the minimum size required by the experiment, we can choose the maximum value in the near field. A wedge with a distance of 6.34 mm can be selected in this experiment.

## 4. Conclusions

According to the principle of ultrasonic Lamb wave nondestructive testing, this paper built a measurement system for liquid monitoring in a closed thin-wall container and studied the internal wedge parameters of the Lamb wave sensor. We first used MATLAB and corresponding programs to draw the phase velocity and group velocity dispersion curves of Lamb waves in aluminum alloy. According to the dispersion curves, we theoretically analyzed and selected the most suitable working mode as A1 when *fd* = 3 MHz·mm and calculated the corresponding ultrasonic incident angle as 27.39°. Then, we verified the correctness of the theory through experiments. Secondly, we studied the wedge thickness. The evaluations showed that, when the vertical distance *L*_1_ from the transmitter sensor to the container wall just avoided the near-field area (9.84 mm), the experimental effect was better, and the received energy decreased with the increase in thickness. When the sound wave propagation distance was an odd multiple of a quarter-wavelength, the received ultrasonic energy was larger. Therefore, when designing a Lamb wave sensor wedge, the inclination angle of the wedge must first be determined according to the material properties of the measured object. When determining the thickness of the wedge, the ultrasonic near-field area should be avoided appropriately. Lastly, it is necessary to ensure that the distance *L*_1_ is an odd multiple of the quarter-wavelength of the ultrasonic wave.

The liquid-level detection method proposed in this paper can realize fixed-point noncontact liquid level measurement, which can be used in aerospace and industrial fields. This paper comprehensively studied the key parameters of the inner wedge of the ultrasonic Lamb waves sensor. The research results can be used to select the best working mode of ultrasonic Lamb waves, while also providing an effective basis for designing the optimal and minimum sizes of wedge blocks.

## Figures and Tables

**Table 1:** Experimental parameters and initial values.

Symbol	Specification	Initial Values
*M_C_*	Container material	Aluminum alloy (Al)
*M_B_*	Wedge material	PMMA
*E*	Al Young’s modulus	70 GPa
*ρ*	Al density	2.7 g/cm3
*v*	Al Poisson’s ratio	0.3
*c_L_*_1_	Ultrasound velocity	2775 m/s
*D_S_*	Transducer diameter	10 mm
*f*_0_	Working frequency	1 MHz, 500 kHz
*T*	Experimental temperature	20 °C

**Table 2:** Evaluations of different inclination angles.

Angle	Liquid Level	*V*_1_ (mV)	*V*_2_ (mV)	*V*_3_ (mV)	$\bar{V}\;(\mathrm{mV})$	|∆*E*| (mV)	*V_d_* (mV)
0°	12 cm	116	112	116	114.67	1.78	182.00
	Air	296	298	296	296.67	0.89	
15°	12 cm	356	352	356	354.67	1.78	310.67
	Air	652	656	688	665.33	15.11	
30°	12 cm	784	832	872	829.33	30.22	680.67
	Air	1450	1500	1580	1510	46.67	
45°	12 cm	172	184	190	182	6.67	170.00
	Air	338	356	362	352	9.33	
60°	12 cm	592	616	600	602.67	8.89	312.00
	Air	912	920	912	914.67	3.56	

Angle: wedge block inclination angle; *V*_1_: the first measured voltage value; *V*_2_: The second measured voltage value; *V*_3_: the third measured voltage value; $\bar{V}$: the average voltage value; |∆*E*|: the average deviation; *V_d_*_:_ the difference value.

**Table 3:** Wedge block parameters and initial values.

Specification	Initial Values (mm)											
Sound Field	Near-Field Region					Far-Field Region						
Height	**0**	**1**	**2**	**3**	**4**	5	6.2	7.11	8.53	9.66	11.04	12.21
*L*_1_	**4**	**5.2**	**6.34**	**7.3**	**8.68**	9.84	11.23	12.6	14.04	15.42	16.81	18.21
Wavelength	-					0	+1/2 *λ*	+*λ*	+3/2 *λ*	+2 *λ*	+5/2 *λ*	+3 *λ*
Material	PMMA											
Width	20											
Angle *α*	30°											

**Table 4:** The relationship between wedge block parameters and wavelengths.

Specification	Initial Values (mm)					
Height	6.2	7.11	8.53	9.66	11.04	12.21
*L*_1_	11.23	12.6	14.04	15.42	16.81	18.21
*L*_1_/(1/4*λ*)	8.09	9.08	10.11	11.11	12.11	13.12
*Λ* = 5.55 mm	Even multiple	Odd multiple	Even multiple	Odd multiple	Even multiple	Odd multiple

## References

[1] Zakaria Z., Idroas M., Samsuri A., Adam A.A. (2017). Ultrasonic instrumentation system for Liquefied Petroleum Gas level monitoring. J. Nat. Gas Sci. Eng..

[2] Yin L., Qin Y., Liu X.-W. (2017). A new interface weak-capacitance detection ASIC of capacitive liquid level sensor in the rocket. Mod. Phys. Lett. B.

[3] Lata A., Kumar B., Mandal N. (2018). Design and development of a level transmitter using force resistive sensor as a primary sensing element. IET Sci. Meas. Technol..

[4] Kumar B., Mandal N. (2016). Study of an Electro-Optic Technique of Level Transmitter Using Mach-Zehnder Interferometer and Float as Primary Sensing Elements. IEEE Sens. J..

[5] Wang N., Zhang Y., Jin B., Wang Y., Zhang M. (2017). Quasi-Distributed Optical Fiber Sensor for Liquid-Level Measurement. IEEE Photonics J..

[6] Berketis K., Tzetzis D., Hogg P. (2009). Noncontact ultrasonics used for impact damage detection on long-term water-immersed GFRP composites. Int. J. Microstruct. Mater. Prop..

[7] Zhang M., Li S. A method of the untouched ultrasonic liquid level measurement with high precision. Proceedings of the 2010 International Conference on Computer Application and System Modeling (ICCASM 2010).

[8] Jun L. (2007). Impact of pressure and temperature upon ultrasonic velocity in two sorts of hydraulic oil. Tech. Acoust..

[9] Haohao H., Junqiao X. A method of liquid level measurement based on ultrasonic echo characteristics. Proceedings of the 2010 International Conference on Computer Application and System Modeling (ICCASM 2010).

[10] Guangjian G. (2012). Examination of ultrasonic Lamb waves for detection of flaws in the bottom plate of oil tank. Appl. Acoust..

[11] Rai A., Mitra M. (2022). A transfer learning approach for damage diagnosis in composite laminated plate using Lamb waves. Smart Mater. Struct..

[12] Lowe P.S., Scholehwar T., Yau J., Kanfoud J., Gan T.H., Selcuk C. (2017). Flexible Shear Mode Transducer for Structural Health Monitoring Using Ultrasonic Guided Waves. IEEE Trans. Ind. Inform..

[13] Moll J., Rezk-Salama C., Schulte R., Klinkert T., Fritzen C.-P., Kolb A. (2011). Interactive simulation and visualization of lamb wave propagation in isotropic and anisotropic structures. J. Phys. Conf. Ser..

[14] Spratt W.K., Vetelino J.F. Torsional acoustic waveguide sensor for temperature and liquid level. Proceedings of the Torsional Acoustic Waveguide Sensor for Temperature and Liquid Level.

[15] Balasubramaniam K., Subhash N.N. (2014). Fluid level sensing using ultrasonic waveguides. Insight-Non-Destr. Test. Cond. Monit..

[16] Yu L., Tian Z. (2015). Case study of guided wave propagation in a one-side water-immersed steel plate. Case Stud. Nondestruct. Test. Eval..

[17] Shen Z., Chen S., Lei Z., Yao K., Tan C.Y. (2017). Direct-Write Piezoelectric Ultrasonic Transducers for Non-Destructive Testing of Metal Plates. IEEE Sens. J..

[18] Zamanov A.D., Agasiyev E.R. (2011). Dispersion of lamb waves in a three-layer plate made from compressible materials with finite initial deformations. Mech. Compos. Mater..

[19] Kim Y., Han J. (2010). Development of hybrid one-dimensional finite elementanalytical method for analysis of lamb wave propagation characteristics in composit panels. J. Acoust. Soc. Am..

[20] Wilcox P., Lowe M., Cawley P. (2001). The effect of dispersion on long-range inspection using ultrasonic guided waves. NDT E Int..

[21] Matsuda Y., Yoshioka M., Uchida T. (2014). Absolute Hydrophone Calibration to 40 MHz Using Ultrasonic Far-Field. Mater. Trans..

[22] Liu J., Li G. (2012). Frequency and Temperature Characteristics of an Ultrasonic Method for Measuring the Specific Gravity of Lead-Acid Battery Electrolyte. Jpn. J. Appl. Phys..

[23] Nikolaevtsev V.A., Suchkov S.G., Selifonov A.V., Suchkov D.D., Suchkova S.M. Efficiency of lamb wave excitation by wedge-shaped ultrasonic transducer. Proceedings of the 2016 13th International Scientific-Technical Conference on Actual Problems of Electronics Instrument Engineering (APEIE).

[24] Wilcox P.D., Dalton R.P., Lowe M.J.S. (1999). Mode and Transducer Selection for Long Range Lamb Wave Inspection. Key Eng. Mater..

[25] Lijian Y., Haice S.U., Songwei G., Yanhao X. (2015). A Method for Increasing Efficiency of Electromagnetic Ultrasonic Lamb Wave Transducer at Tank Bottom Plate. Oil & Gas Storage and Transportation. http://en.cnki.com.cn/Article_en/CJFDTotal-YQCY201508008.htm.

[26] Rose J.L., Nagy P.B. (2000). Ultrasonic Waves in Solid Media. J. Acoust. Soc. Am..

[27] Deighton M.O., Gillespie A.B., Pike R.B., Watkins R.D. (1981). Mode conversion of Rayleigh and Lamb waves to compression waves at a metal-liquid interface. Ultrasonics.

[28] Breakey D.E.S., Jordan P., Cavalieri A.V.G., Olivier L., Rodríguez D. Near-field wavepackets and the far-field sound of a subsonic jet. Proceedings of the 19th AIAA/CEAS Aeroacoustics Conference.

[29] Lukosevicius A., Jurkonis R., Jurkonis R. (2014). Ultrasonic near field in lossy media: Method of simulation. Ultrasound.

[30] Chen Q., Wang X., Li M., Mao J. (2011). Sound field of an electromagnetic acoustic transducer. Chin. J. Acoust..

[31] Liu J.-x., Wang Z.-q., Li G.-f., Wang N.-h. (2011). Acoustic method for obtaining the pressure reflection coefficient using a half-wave layer. Ultrasonics.

[32] Gao W., Liu W., Hu Y., Wang J. (2020). Study of Ultrasonic Near-Field Region in Ultrasonic Liquid-Level Monitoring System. Micromachines.

[33] Liu Y., Li H.J. (2013). Wave reflection and transmission by porous breakwaters: A new analytical solution. Coast. Eng..

[34] Pilarski A., Rose J.L. (1992). Lamb wave mode selection concepts for interfacial weakness analysis. J. Nondestruct. Eval..

